# Notes and News

**Published:** 1890-04-12

**Authors:** 


### Notes and News.

Collected and Communicated.

A case of glanders occurred lately at University College Hospital. A coal [porter, aged 23, was admitted on the 24th ult. by Dr. Cyril Jecks, who, on examination, found him to be suffering from glanders. The man got gradually weaker, and died on the 31st; he was unable to state how he contracted the disease.

We are glad to see that Princess Christian is in better health, and is expected back to England in a few days. There are several matters in the nursing world which require the immediate attention of Princess Christian, who will doubtless act on the advice of the Duke of Westminster, the trusted adviser of the Queen on all such questions.

The Pope is going to "reform" the Benedictine order, that is, is going to insist on all the old rules which hid the brethren and sisters utterly from the world, being again enforced. The Brothers of Charity in Austria have pleaded against the "reform," as they have numerous large hospitals, the one in Vienna holding nine hundred beds, and their young members study at the Universities and become doctors and physicians. If the new rules are enforced, this useful work will be hindered, and in a great measure stopped, as the brothers must now cease intercourse with the world, and attend exclusively to their religious obligations.

The Paris correspondent of the *Daily News* gives a pathetic picture of an Easter entertainment given to the epileptic children of the Bicêtre Asylum. A little marionette theatre was set up, and it is to be feared the thousand spectators were much perplexed by what was said and done in the little theatre. They had never heard of Boulanger, the Eiffel Tower, and many other topics familiar to the ordinary Parisian. However, they awoke to more intelligent apprehension when the well-known "The Dentist's Apprentice" was being played. This is in the style of Punch and Judy. The characters whack each other soundly, and whenever one belaboured the other the idiots saw the fun and were convulsed with laughter, and there was hardly a blank face. The impression of gaiety seemed, however, to die out wholly the instant the curtain fell. Another interesting Easter festival at Paris was the performance of Gounod's Mass, by a choir of blind children. They sang beautifully, reading their scores by means of their fingers.

The annual report of Boscombe Hospital appears with a large plan appended showing the proposed alterations which the increase in the work of the hospital make absolutely necessary. The report is well drawn up, very full, and very interesting. This hospital certainly marches with the times, and the committee are to be congratulated. Mr. G. E. Weary (house surgeon) and Miss Fletcher (matron) conduce greatly by their energy to the success of the institution.

The explanation of last year's epidemic of diphtheria at Alma Square and the neighbourhood is now fully accounted for by Dr. Wynter Blyth, who tells us that the epidemic derives its interest neither from the number of persons attacked nor from any unusual fatality, but from the circumstance that the causes were of a distinctly preventable kind. The primary source of the mischief appears to have been the defective condition of the main drain. Dr. Blyth reports that this drain, laid at only two feet and a-half below the pavement, had not fall enough to empty itself. As a consequence soil collected in the glazed pipes, and the joints not having been cemented, or apparently clayed up, the drainage of the houses had been escaping until the ground around them had become perfectly black. It obviously needed nothing but a slight leakage in the water company's service pipes laid down in this soil to produce serious contamination, and such leaks have been found.

The annual meeting of subscribers to the Savernake Hospital was held on March 28, Mr. W. H. Long, M.P., in the chair. The report says that "During the year 1889, the number of admissions has been 213, being 17 more than in the previous year. These came from 54 different parishes. Of these 126 were discharged cured, 62 relieved, and five died. The income for the year was £797 14s. 1d., the expenditure £723 14s. 9d., showing a balance on the year of £73 19s. 4d.; and, after deducting the adverse balance of 1888 (£21 17s. 3d.), leaving a balance in hand of £52 2s. 1d. During the past year a new special fund has been raised to meet the expense of connecting by a telephone the hospital with the houses of the medical officers. The committee have to record with deep regret the resignation of the Rev. J. E. Gordon Bond as honorary secretary, and they desire to place on record their great appreciation of the many benefits the institution received from him. For eleven years he had discharged the duties of secretary and chaplain."

The Mayor presided at the annual meeting of the Queen's Hospital, Birmingham, and in moving the adoption of an excellent report, said the income had been £1,200 more than in the previous year, and almost for the first time in the history of the hospital their accounts were now square. There had been a large increase in the number of out-patients treated during the year, and a decrease in the in-patients was due to the fact that owing to important alterations that had been going on during the year several wards had been closed. Those alterations were now happily completed. The improvement in the accommodation for the nurses he regarded as most important. The nurses when off duty would occupy a building specially erected for the purpose, in which they would be entirely removed from the atmosphere of the hospital, and in which comfortable sitting-rooms had been provided. As an experiment they intended to appoint a Nursing Committee for twelve months, and before nurses were finally appointed the probationers would have their antecedents more closely investigated than had been the case hitherto. Another improvement had been the extension of the pathological department. As an old member of the committee he could not conclude without refering to the fact that their late senior physician, Sir James Sawyer, was now in a sense retiring into private life, so far as concerned the actual work of the hospital.

The following vacancies are announced this week, the amount of the salary in each case being given after the name of the institution :—

**Resident Medical Officers.**

Chelsea Hospital for Women, Medical Officer—£80, board, etc.
Holborn Union, Assistant Medical Officer—£100, board, etc.
Paddington Green Children's Hospital, House Surgeon—£50, board, etc.
Female Lock Hospital, Harrow Road, House Surgeon—£100, board, etc.
Birmingham General Dispensary, Surgeon—£150, rooms, etc.
Leeds Infirmary, Two House Physicians—Board, etc.
Leeds Infirmary, Two House Surgeons—Board, etc.
Colney Hatch Asylum, Medical Superintendent—£1,000.

**Non-Resident Medical Officers.**

Sun Life Assurance Society, Second Medical Officer.
Goole Friendly Society, Medical Attendant—£230.
Owens College, Manchester, Demonstrator in Materia Medica and Pharmacy—£100.
City of London Hospital, Pathologist.
Islands of Yell and Fetlar, Medical Officer—£70.

The Mayor of Shrewsbury presided at the annual meeting of the local dispensary last week. The aggregate number of enrolled members up to the 31st of December last was 21,536, of which number 1,009 entered in 1889. The weekly average of patients was 1,021, as against 1,044 in 1888. The exceptional amount of illness during the last six months has much increased the labours of the medical officers, for Shrewsbury, like the rest of the world, has been visited by influenza. A very favourable balance-sheet is issued, and the dispensary can be congratulated on the success of its labours.

Hypnotism is becoming more popular, and is daily attracting more attention from medical men. At Leeds some dental surgeons have been extracting teeth, and surgeons have performed operations on patients hypnotised by Dr. Milne Bramwell. At the Royal Aquarium just now there is a clever exponent of this art, who nightly amuses the crowd by the power he exercises on some half-dozen victims. In *The Forum* is an article, by no less an authority than Dr. Charcot himself, on the possibilities of a criminal use of the hypnotic influence. He proves that a limited number only are capable of being hypnotised—"nervous creatures capable of becoming hysterical, if not actually hysterical, at the beginning of the experiments." Men, even though hysterical, are seldom and only with difficulty hypnotisable. Then, most important of all, persons may have, even in the hypnotic state, the capability of resisting a suggestion that is not congenial. Still it is evident that under certain circumstances hypnotism might not be an unmitigated blessing, and that physicians are right in approaching the subject with caution.

On Saturday last the public sanitary inspectors of Great Britain held a meeting, and a paper was read by Mr. F. T. Poulton on the somewhat philosophical subject of "Social Environment." Mr. Poulton did not treat the subject from the Spenserian point of view, however, but took it in its more practical form. He emphasized the necessity of proper sites for building purposes being selected, and of keeping down of earth miasma, contending that families living in houses built on damp, heavy land, lately used as market gardens often suffered from diphtheria and other zymotic diseases. With regard to the water supply, he advocated a compulsory constant service being established in all thickly-populated districts, which could be depended upon in case of fire, laid on at a high pressure, and always obtainable in all parts of the house. As to the disposal of the dead, he declared that, from a sanitary point of view, our system of burial was perhaps the most vile that it was possible to conceive, and he urged the desirability of an advance even on cremation, by which the mortality, which was very high in the neighbourhoods of graveyards, would be largely reduced.

The *Gazette* of India comments strongly on the increase of opium eating at Lucknow. One shop in Lucknow, opened shortly after the mutiny, not only still exists, but the sum realized by its lease has increased more than eight times within the last seven years. Its daily frequenters average 400. The great mass of opium-eaters belong to the lower classes, and opium-smoking is almost confined to the lowest of the Mohammedan class living in Lucknow. Yet cases are not unknown in India in which Europeans have fallen slaves to this degrading habit.

The public interest in Giovanni Succi, who is attempting a forty days fast at the Royal Aquarium, is scarcely comprehensible. To go and stare at a half-starved man seems poor sport, and one which can unfortunately be indulged in free of charge any day at the entrance to the docks. There is some scientific interest in Succi's attempt, especially as the case is being carefully watched by the students of the Westminster Hospital. In spite of loss of weight, up to Wednesday, Succi was cheerful and retained his muscular strength; he had then completed twenty-three days of his fast, and was confident of bringing it to a successful termination.

